# PKCα mediated induction of miR-101 in human hepatoma HepG2 cells

**DOI:** 10.1186/1423-0127-17-35

**Published:** 2010-05-06

**Authors:** Chao-Wei Chiang, Yi Huang, Ka-Wai Leong, Lih-Chyang Chen, Hua-Chien Chen, Shu-Jen Chen, Chen-Kung Chou

**Affiliations:** 1 Institute of Microbiology & Immunology, National Yang-Ming University, Taipei, Taiwan; 2 Department of Life Science, Graduate Institute of Basic Medical Science, Chang Gung University, Tao-Yuan, Taiwan; 3 Chang Gung Molecular Medicine Research Center, Chang Gung University, Taoyuan, Taiwan; 4 Genomic Core Laboratory, Molecular Medicine Research Center, Chang Gung University, Taoyuan, Taiwan

## Abstract

**Background:**

Protein Kinase C (PKC) is a serine/threonine kinase that involved in controlling of many cellular processes such as cell proliferation and differentiation. We have observed previously that TPA (12-O-tetradecanoylphorbol 13-acetate) induces cell cycle arrest in G0/G1 phase in human hepatoma HepG2 cells. However, is there any miRNA involved in PKCα mediated cell growth arrest is still unknown.

**Methods:**

We first surveyed 270 miRNA expression profiles in 20 pairs of human hepatoma tissues. We identified 11 up-regulated and 23 down-regulated miRNAs (FDR < = 0.01; fold-change > = 2) in human hepatoma tissue after Student's *T*-test and Mann-Whitney rank test. We then examined miRNAs expression profile in TPA treated HepG2 cells. Two miRNAs, miR-101, and miR-29c, were shown to be significantly down regulated in human hepatoma tissues and induced over 4-fold in HepG2 cells under TPA treatment.

**Results:**

In this study, we examined TPA regulated miRNA expression profile in human hepatoma HepG2 cells. We identified two miRNAs, 101 and 29c, were induced by TPA and down regulated in human hepatoma tissues suggest that they might play as tumor suppressor gene and in tumor formation of HCC. Since induction kinetics of miR-101 by TPA was much faster than miR-29c suggests that the induction of miR-101 may be the primary response of TPA treatment. We then further investigated how miR-101 was regulated by TPA. MiR-101 targets two subunits of PRC2 complex, enhancer of zeste homolog 2 (EZH2) and EED, and was shown to play as a tumor suppressor gene in human prostate, breast and liver cancers. The target sequence of miR-101 located in the 3' UTR of both EZH2 and EED's mRNA was identified by bioinformatic analysis and was validated by reporter luciferase activity assay. Then we showed that TPA not only up regulated miR-101 expression, but also reduced protein level of EZH2, EED and H3K27me3 in HepG2 cells. Using lenti-virus-mediated shRNA to knockdown endogenous PKCα expression, we observed that TPA induced growth arrest, elevation of miR-101 and reduction of EZH2, EED and H3K27me3 proteins were all PKCα dependent. Specific inhibitor of ERK completely blocked TPA induced miR-101 expression.

**Conclusions:**

Therefore, this is the first time to show that PKCα and ERK pathway play important role to activate miR-101 expression, reduce PRC2 complex and H3K27me3 level. This epigenetic regulatory pathway may represent a novel mechanism of carcinogenesis and deserve further investigation.

## Background

MicroRNAs (miRNAs) have been shown to regulate gene expression either at the post-transcriptional or at the translational levels [1]. Recent analysis of global miRNA expression profile in various cancer tissues has revealed significant alteration of a specific set of miRNA in breast, lung, pancreas tumors and leukemia [2,3]. The cause and consequences of miRNA dysregulation in cancer has been intensively reviewed recently [4]. MicroRNAs have also been shown to play important role in cell cycle control [5]. For example, members of the miR-290 cluster were shown to regulate the G1/S phase transition in embryonic stem cell [6]. Overexpression of miR-203 was shown to induce the differentiation of human keratinocytes [7,8]. However, very little is known about how miRNA itself was regulated under various physiological conditions.

PKC is a member of serine/threonine kinase whose isoforms have been shown to be involved in a number of cellular processes, including cell proliferation, apoptosis, invasion and migration [9,10]. Various PKC isoforms have been identified, including the conventional PKCs (cPKC-α, cPKC-βI, cPKC-βII, and cPKC-γ), novel PKCs (nPKC-δ, nPKC-ε, and nPKC-η), and atypical PKCs (aPKCζ) [11]. *In vitro *and *in vivo *studies clearly documented that PKC signaling has the potential to regulate cell proliferation [12,13]. Previous studies have shown that TPA activates protein kinase C alpha and induces growth arrest of human hepatoma HepG2 cells [14]. However, whether there is any miRNA involved in PKCα-mediated cell growth arrest is still unknown.

MiR-101 was shown to promote apoptosis and suppress FOS oncogene expression in human hepatoma cells and to act as tumor suppressor gene in carcinogenesis of human hepatoma [15,16]. The targets of miR-101 include EZH2 and EED, two key component of PRC2 complex. PRC2 is responsible for genome wide methylation of histone 3 lysine 27 [17]. Therefore, down regulation of miR-101 in HCC may increase PRC2 complex, enhance methylation of histone H3 lysine 27 at specific genome loci and epigenetically regulate gene expression at genome wide level.

In this study, we examined TPA regulated miRNA expression profile in human hepatoma HepG2 cells and discovered that miR-101 was induced by TPA in HepG2 cells. We also showed the induction of miR-101 by TPA is PKCα and ERK dependent. This result opens a new direction to study molecular mechanism of dysregulation of miRNA expression in human HCC in the future.

## Methods

### Plasmid constructs and cell lines

Lentiviral plasmids of Clone ID: TRCN0000001692 encoding a shRNA targeting region of PKCα mRNA were obtained from The RNAi Consortium (TRC), National RNAi Core Facility, Academia Sinica, Taiwan. The 3'untranslated regions of EED (1~211) and EZH2 (1~263) were prepared by PCR using NPC-TW02 cells cDNA and cloned into MluI/SpeI sites of pMIR-REPORT™ (Ambion, Austin, TX). MiR-lacZ 5'-TGCTGAAATCGCTGATTTGTGTAGTCGTTTTGGCCACTGACTGACGACTACACATCAGCGATTT-3' cloned into SmaI/NcoI sites of pLenti-6.4 (Invitrogen, Taiwan). MiR-101-1:5'-TGCCCTGGCTCAGTTATCACAGTGCTGATGCTGTCTATTCTAAAGGTACAGTACTGTGATAACTGAAGGATGGCA-3' and miR-101-2: 5'-ACCACCATTCTTCAGTTATCACAGTACTGTACCTTTCAGATATACAGCATCGGTACCATGATAACCGAAAAAGGACAGT-3' were cloned into SmaI/XmaI sites of pLenti-6.4. HEK 293T, HEK 293 and HepG2 cells were obtained from American Type Culture Collection (Rockville, MD). Cells were grown in Dulbecco's modified Eagle's medium (DMEM; Invitrogen, Taiwan) supplemented with 10% (v/v) fetal bovine serum, 2 mM L-glutamine, penicillin (1000 U/ml), and streptomycin (50 μg/ml) with 5% CO2 at 37°C.

### Cell transfection and antibodies

Transfection of plasmid DNA into cultured cell was performed using the standard calcium phosphate precipitation method [18]. Cells were plated in 12-well dishes with 5 × 10^5 ^cells/well 24 hours before transfection. Anti-β-actin (AC-15) was purchased from Sigma (St. Louis, MO). Anti-EED was purchased from Upstate Biotechnology. Anti-EZH2 (AC22), and anti-Tri-Methyl-Histone H3 (Lys27) (C36B11) antibodies were purchased from Cell Signaling Technology (Beverly, MA). Anti-PKCα (C-20), Anti-PKCε (C-15) were purchased from Santa Cruz Biotechnology (Santa Cruz, CA). Anti-PKCδ was purchased from Transduction Laboratories (Lexington, KY). TPA (12-O-tetradecanoylphorbol 13-acetate) was purchased from Sigma (St. Louis, MO). Precursors of respective miR-101 and negative controls were purchased from Ambion (Austin, TX).

### Immunoblotting analysis

Cells were washed with ice-cold PBS twice and lysed in 500 μl of chilled RIPA buffer (50 mM Tris-HCl pH 7.4, 150 mM NaCl, 1 mM PMSF, 1 mM EDTA, 1% Triton X-100, 1% Sodium deoxycholate, 0.1% SDS) with protease inhibitor cocktail from Roche Diagnostics Ltd (Lewes, U.K). The cell debris was removed by centrifugation for 10 min at 14000 rpm in an Eppendorf microcentrifuge. The supernatant was added with SDS sample buffer (100 mM Tris, 25% glycerol, 2% SDS, 0.01% bromophenol blue, pH 6.8) containing 5% β-mercapto-ethanol and boiled for 10 min. Whole cell lysates were resolved by SDS-polyacrylamide electrophoresis. After transferred to nitrocellulose membranes (PerkinElmer Life science), the membranes were blocked in 5% non-fat milk/TTBS (25 mM Tris-HCl, pH 7.4, 137 mM NaCl, 3 mM KCl, and 0.2% Tween 20) followed by incubation with the indicated primary antibodies. Membranes are then incubated with horseradish peroxidase-conjugated secondary antibodies and levels of proteins of interest were detected by ECL chemi-luminescence reagents as described (Visual Protein Biotechnology Corp.).

### RNA extraction and quantitative reverse transcription PCR (Q-PCR)

Total RNA was prepared using TRIzol reagent (Invitrogen) according to the manufacturer's protocol. For quantitative measurement of miRNA, stem-loop RT-qPCR assay was performed as described [19]. Briefly, 1 μl of diluted RT product was used as template for a 10 ml PCR. The PCR reaction mixture contains 1× SYBR Master Mix (Applied Biosystem, Foster City, CA, USA), 200 nM miRNAs-specific forward primer, and 200 nM universal reverse primer. The condition for Q-PCR is 95°C for 10 min, followed by 40 cycles of 95°C for 15 s and 63°C for 32 s, and a dissociation stage [20]. For mRNA Q-PCR reaction, the following PCR conditions were used: 95°C for 10 min, followed by 45 cycles of 95°C for 15 s and 60°C for 1 min, and a dissociation stage [20]. An ABI Prism 7500 Fast Real-Time PCR system (Foster City, CA, USA) was used for Q-PCR reactions. The threshold cycle (C_t_) and relative quantification (RQ) were calculated by using the ABI 7500 SDS 1.3.1 software. The primers used in Q-PCR are shown in Table 1.

**Table 1:** Q-PCR primers.

Name	Forward primer	Reverse primer
hsa-miR-101	CGGCGGTACAGTACTGTGATAA	Universal stem-loop primer*
hsa-miR-29c	CGGCGGTAGCACCATTTGAAAT	
hsa-miR-122	CGGCGGTGGAGTGTGACAATGG	
hsa-miR-16	CGGCGGTAGCAGCACGTAAATA	
B2M	AGGACTGGTCTTTCTATCTCT	TTCATCCAATCCAAATGCGG

* The universal stem-loop primer: CTGGTGTCGTGGAGTCGGCAATTC

### Data processing

The threshold cycle (C_t_) is defined as the cycle number at which the change of fluorescence intensity crosses the threshold of 0.2. The raw C_t _data were converted to 39-C_t _after normalized by global median normalization before further analysis. Student's *T*-test was performed to identify differentially expressed (DE) miRNAs (p < 0.05) and those miRNAs with a fold-change less than 2 were then filtered. For mRNA expression, the average C_t _of β-2-microglobulin (B2M) was subtracted from the raw C_t _value to obtain ΔCt (dC_t_). Because any Ct value greater than 40 is considered undetectable, the experimentally normalized dC_t _values were converted to 39-Ct and used to represent the expression level of human mRNA transcripts. The DE genes were identified by one-way ANOVA and calculate q-value (false discovery rate). Genes with more than 10% FDR and less than 1.5-fold changes were filtered out. Partek^® ^Genomics Suite (version 6.4, St Louis, MO, USA) was used for all statistical analyses.

### Luciferase reporter assay

Cells were co-transfected microRNA expression vector pLenti-6.4 containing miR-101-1, miR-101-2, or miR-LacZ, with pMIR-REPORT constructs of EED-3'UTR(1~221) and EZH2-3'UTR(1~263), and Rous sarcoma virus-β-galctosidase vector to monitor transfection efficiency. Forty-eight hours after transfection, cell extracts were analyzed for luciferase and β-galactosidase activities using the Dual-Light Kit (Tropix) according to the manufacturer's instruction. Luciferase activity was normalized to β-galactosidase activity and expressed as fold stimulation relative to vector-transfected cells. Results shown are averages of three separate experiments performed in triplicate. Values are expressed as means ± s.d.

### Cell proliferation assay

MTT [3-(4,5-dimethylthiazol-2-yl)-2,5-diphenyl-2*H*-tetrazolium bromide; Sigma] reduction assays were performed as previously described [21]. HepG2 cells were seeded in 24-well plates overnight in DMEM containing 10% (v/v) FBS. Before colorimetric determination of MTT reduction, MTT was added to a final concentration of 0.5 mg/ml, and incubation was continued for another 4 h before adding the cell lysis buffer [20% (w/v) SDS and 50% (v/v) *N*,*N*-dimethylformamide, pH 7.4]. The colorimetric determination of MTT reduction was performed at the wavelength of 570 nm.

## Results

### TPA-induced growth arrest in HepG2 is PKCα-dependent

**Figure 1:**
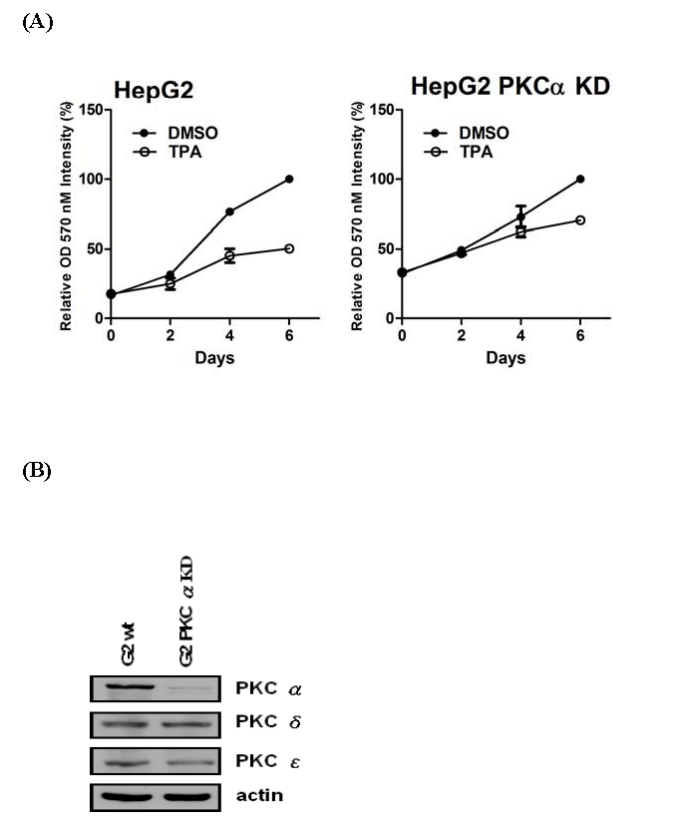
**TPA-induced growth arrest in HepG2 is PKCα-dependent**. (A) 1 × 10^5 ^of parental HepG2 and PKCα knockdown HepG2 cells were seeded in 24 well plates. After culturing in serum-free medium for 24 hrs, cells were treated with TPA 100 nM in serum-free medium for indicated time before MTT analysis. The cell proliferation rate were analyzed using Day6 as 100%. Results shown are averages of three independent experiments performed in triplicates. (B) The expression profile of different PKC isoforms in parental HepG2 and HepG2 PKCα knockdown stable lines were examined by Western blot analysis.

Previous studies have shown that PKCα may play an important role in TPA-induced growth arrest in HepG2 cells [14]. To re-examine whether the PKCα is required for TPA-mediated cell arrest in HepG2 cells, we knocked down the expression of endogenous PKCα in HepG2 cells using a lentiviral-based PKCα shRNA. We found that TPA induced cell arrest in HepG2 cells was largely abolished in PKCα knockdown HepG2 cells (Fig. 1A). The lentiviral-based PKCα shRNA specifically knocked down only PKCα and has no effect on the expression of two other PKC isoforms, PKCδ and PKCε, in the HepG2 cells (Fig. 1B).

### Identification of key regulatory miRNA in TPA induced growth arrest in HepG2 cells

**Figure 2:**
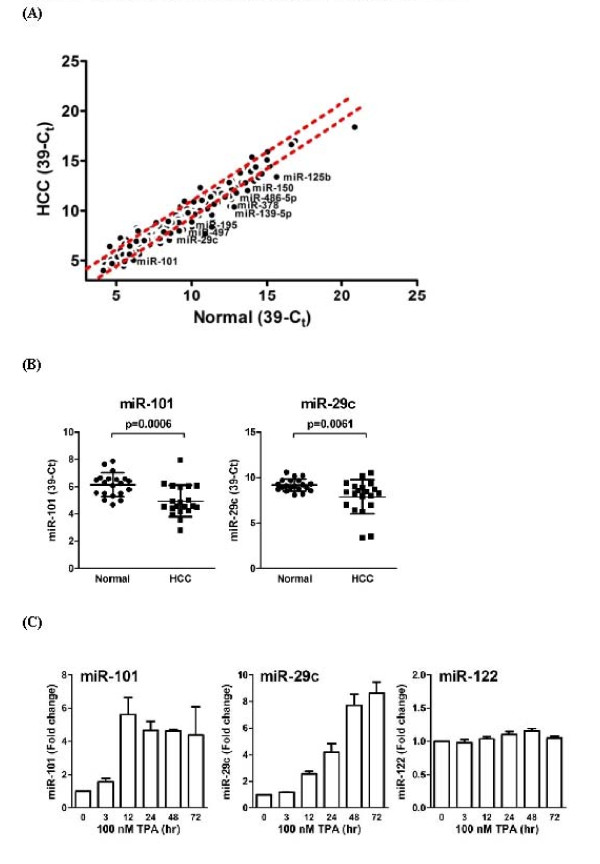
**Identification of key regulatory miRNA in TPA induced growth arrest in HepG2 cells**. (A) Expression levels of 270 miRNAs in 20 pairs of human HCC tissues. The labeled miRNAs were inversely modulated in TPA-treated HepG2 cells and dotted lines indicate the 2-fold change threshold. Expression levels of miRNA were presented as 39-Ct. (B) Expression levels of miR-29c and miR-101 in 20 pair of human HCC tissues and their adjacent normal tissues. Expression levels of miRNA were presented as 39-Ct. p-values were calculated using T-test. (C) Time-dependent changes in miR-101, miR-29c and miR-122 expression levels. HepG2 cells were treated with 100 nM TPA for indicated time periods and the total RNAs were collected for stem-loop RT-qPCR. Expression levels of miR-101, miR-29c and miR-122 were normalized to miR-16 and expressed as fold-change using time 0 as baseline.

To identify miRNAs with novel regulatory activity, we hypothesized that any miRNA plays key regulatory role in TPA-induced cell growth arrest should also shown altered expression pattern in human hepatoma tissues. We first surveyed the expression profile of 270 human miRNAs in 20 pairs of human hepatoma tissues. Using Student's *T*-test and Mann-Whitney rank test, we identified 11 up-regulated and 23 down-regulated miRNAs (FDR ≤ 0.01; fold-change ≥ 2) in human hepatoma tissue (Figure 2A). We then examined the miRNA expression profile in TPA treated HepG2 cells. Two miRNAs, miR-101, and miR-29c, were found significantly down regulated in human hepatoma tissues and induced over 4-fold in HepG2 cells upon TPA treatment (Fig 2B; Table 2).

**Table 2:** Fold-change of inversely modulated miRNAs in HCC samples and TPA-treated HepG2 cells.

Names	Chromosome location	Seed seq.	Seed Family	HCC P-value	HCC (T/N)^a^	TPA (T/C)^b^
hsa-miR-101	1p31.3 9p24.1	ACAGUAC	miR-101	2.30E-04	-2.17	13.36
hsa-miR-29c	1q32.2	AGCACCA	miR-29	5.90E-03	-2.31	7.26

a, tumor versus normal fold-change; b, TPA versus DMSO (control) fold change

The induction kinetics of both miR-101 and miR-29c in HepG2 cells after TPA treatment were examined. Interestingly, these two miRNAs showed completely different induction kinetics after TPA treatment in HepG2 cells. MiR-101 was rapidly induced by TPA at 3 hrs and reached maximum level of induction at 12 hrs. On the other hand, miR-29c only showed slight induction after 12 hrs and reached maximum level of induction at 48 hrs. The rapid induction of miR-101 by TPA treatment indicates that the induction of miR-101 may be the primary response of TPA treatment in HepG2 cells (Fig 2C).

### TPA-induced miR-101 and its downstream effects are all PKCα-dependent in HepG2 cells

**Figure 3:**
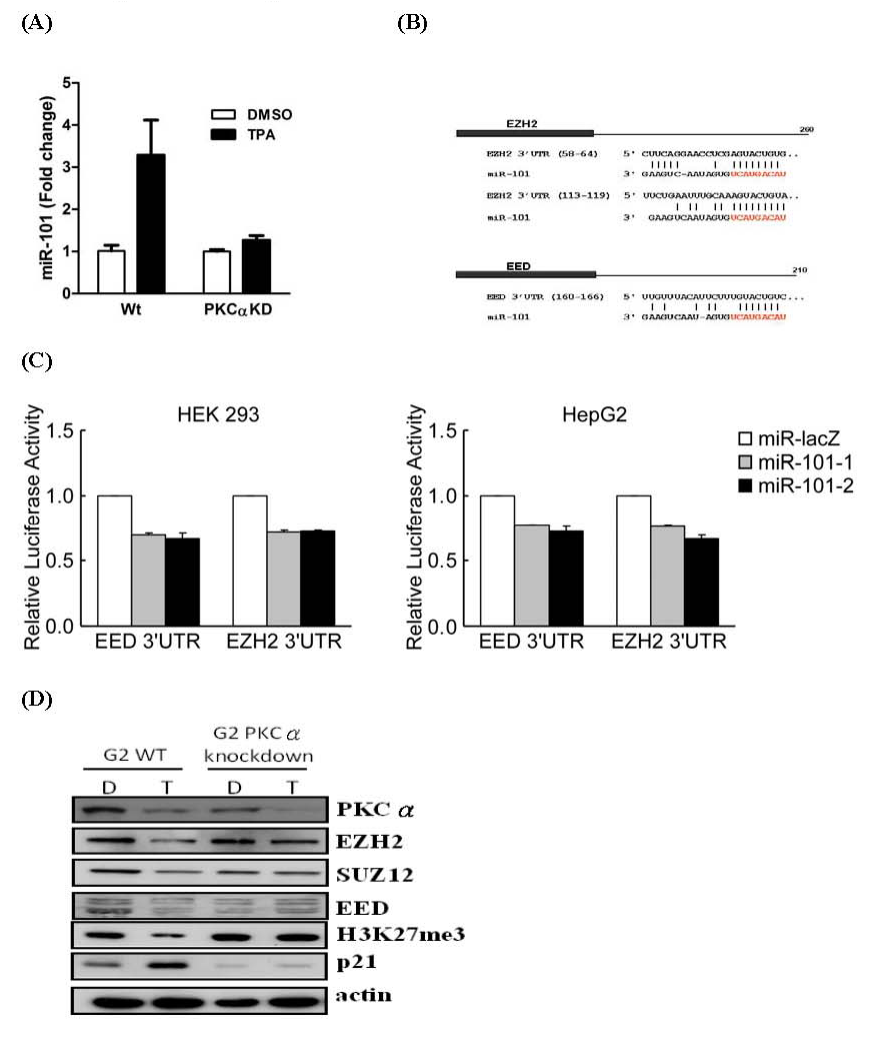
**TPA-induced miR-101 and its downstream effects are all PKCα-dependent in HepG2 cells**. (A) HepG2 and HepG2 PKCα knockdown cells were treated with 100 nM TPA for 48 hours. Expression levels of miR-101 was normalized to miR-16 and expressed as fold change using DMSO-treated sample as baseline. (B) Alignment of miR-101 sequence and the predicted miR-101 target sites in the 3'UTR of EZH2 and EED. (C) HEK293 and HepG2 cells were co-transfected either pMIR-REPORT constructs of EED-3'UTR(+1~+ 211) and EZH2-3'UTR(+1~+263) 10 ng with 4 μg of miR-LacZ, miR-101-1, and miR-101-2 for 48 hours. Forty-eight hours after transfection, cells were harvest for luciferase activity analysis. Results shown are averages of three independent experiments performed in triplicate. (D) HepG2 and HepG2 PKCα knockdown cells were treated with 100 nM TPA for 48 hours. Cell lysates were collected for immunoblotting assay using the PKCα, EZH2, SUZ12, EED, p21, and H3K27me3 antibodies.

To study how TPA induced miR-101 expression in HepG2 cells, we examined miR-101 expression in both parental HepG2 and PKCα knockdown HepG2 cells under TPA treatment. As shown in Fig. 3A, TPA induced miR-101 expression was largely abolished in the PKCα knockdown HepG2 cells. This result clearly indicates that TPA-induced miR-101 expression in HepG2 cells is PKCα dependent.

EZH2 and EED are key components of PRC2 complex, a critical epigenetic modulator responsible for genome wide methylation of histone 3 lysine 27. EZH2 and EED have been shown as the target gene(s) of miR-101 [17,22]. To examine the effect of miR-101 on these two targets, we first analyzed and identified the miR-101 recognition sequence in the 3' UTR regions of EZH2 and EED using our in-house TargetScan program (Fig. 3B). There are two predicted miR-101 precursor hairpin structures, miR-101-1 and miR-101-2, in the human genome. Both predicted miR-101 precursors generate identical mature miR-101. MiR-101-1 is an intergenic miRNA gene located in chromosome 1p31.3. miR-101-2 is located in chromosome 9p24.1 and is mapped to the intron of a host gene RCL-1 whose function is still not clear [23]. To experimentally validate that the predicted target sequences of miR-101 can be suppressed by both miR-101-1 and miR-101-2, we cloned these two miR-101 precursor sequences into the pMIR-REPORT™ and perform luciferase activity assay. As shown in Fig. 3C, when the luciferase gene carried 3' UTR region of either EZH2 or EED's transcript, the luciferase activity was inhibited by over-expressing miR-101-1 or miR-101-2 but not by the control miR-LacZ. Similar results were obtained in both HepG2 and HEK293 cells.

If TPA-induced miR-101 expression is PKCα-dependent, all TPA-induced miR-101 down stream effects such as reduced level of EZH2 and EED protein and methylation of histone 3 lysine 27 should also be PKCα-dependent. As shown in Fig. 3D, TPA treatment indeed reduced protein level of EZH2, EED, SUS12 and histone H3 with tri-methylated lysine 27 in parental HepG2 cells. However, in the PKCα knockdown HepG2 cells, TPA has no effect on protein level of EZH2, EED, SUS12 and histone H3 with tri-methylated lysine 27. Cell cycle inhibitor p21 was used as a positive control, since it has been shown before that TPA induced p21 in HepG2 cells is PKCα dependent.

### TPA-induced miR-101 in HepG2 is mediated by ERK signaling pathway

**Figure 4:**
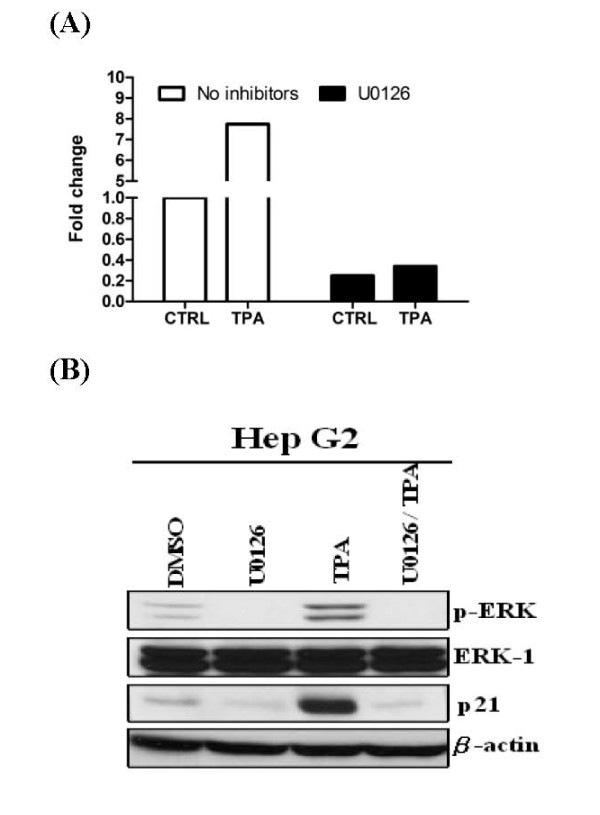
**TPA-induced miR-101 in HepG2 is mediated by ERK signaling pathway**. (A) HepG2 cells were cultured serum-free medium for 24 hrs, pre-treatment of specific MAPK signaling inhibitors U0126 10 μM for 0.5 h, and treated with TPA 100 nM for 8 hrs. Total RNAs were isolated from indicated samples with standard procedures. The expression level of miR-101 was normalized to miR-16 and expressed as fold change using control sample as baseline. Results shown are averages of three independent experiments performed in triplicates. (B) Protein levels of p-ERK, ERK, p21 and β-actin after TPA and ERK inhibitor treatment.

To further identify signaling pathway downstream of PKCα is crucial for TPA induced miR-101 expression, we examined whether the ERK signaling is involved using specific ERK signaling pathway inhibitors. We pretreated HepG2 cells with the specific MAPK inhibitor, U0126 (10 μM), for 30 minutes and then treated cells with TPA for 8 hrs. We found that pretreatment of U0126 completely blocked TPA induced miR-101 expression in HepG2 cells (Fig. 4A). As a control, U0126 blocked TPA induced ERK activation and p21 expression (Fig. 4B).

## Discussion

PKC is a family of phospholipid-dependent serine/threonine kinase and involves in various cellular processes such as cell proliferation, apoptosis, invasion and migration. Previous studies have shown that activation of PKC alpha is required for TPA-induced ERK signaling to trigger gene expressions of p15(INK4b) and p16(INK4a) leading to HepG2 growth inhibition [24]. Recent studies further identified transcriptional factor Snail is up-regulated by PKC alpha and is responsible for inducing p15(INK4b) expression [25,26]. However, any other novel regulator such as miRNA involved in TPA induced growth arrest of HepG2 cells is still unknown. In this study, we demonstrated that TPA-induced ERK signaling pathway in HepG2 cells can up-regulate expression of tumor suppressor gene miR-29c and miR-101.

Several studies have shown that miR-29c is down-regulated in nasopharyngeal carcinomas, chronic lymphocytic leukemia (CLL), and lung cancer which were correlated with up-regulating target genes in extracellular matrix proteins and DNA methyltransferase (DNMT) 3A and -3B [27-29]. Whether miR-29c is also involved in regulating HepG2 cell growth still needs more studies in the future.

MiR-101 recently has been shown to act as an important tumor suppressor gene in various human cancers including prostate and liver cancer [16,17,22]. Two essential components of PRC2 complex, EZH2 and EED, have been shown as target of miR-101 [17]. PRC2 is responsible for genome wide methylation of histone 3 lysine 27 [17]. Therefore, we hypothesized that down regulation of miR-101 in HCC may increase PRC2 complex, enhance methylation of histone H3 lysine 27 at specific genome locus and epigenetically regulate gene expression at genome wide level.

Based on this hypothesis, the first question should be answered is how expression of miR-101 is down regulated during development of human cancers. MiR-101 can be expressed from two genomic loci, miR-101-1 on chromosome 1p31 and miR-101-2 on chromosome 9p24. Both loci produce identical mature miR-101. Therefore, it becomes difficult to differentiate transcriptional regulation of one locus from the other. Only one study convincingly showed that genomic deletion of miR-101 at both loci occurs in a significant number of human prostate cancer and was associated with cancer progression [17].

In our study, we showed unequivocally that activation of PKCα and ERK by TPA can induce expression of miR-101 in HepG2 cells. Our results suggest that in human HepG2 cells the genomic loss may not be responsible for down regulation of miR-101 expression. This conclusion was supported by the results of genomic PCR analysis. No genomic deletion at either miR-101 locus was detected in HepG2 cells (data not shown).

Our study also provided first experimental evidence to show that induction of endogenous miR-101 indeed is accompanied with lower EZH2, EED and SUZ12 level and histone 3 lysine 27 trimethylation in human hepatoma cells. These results indicate that the expressed miR-101 in HepG2 cells is fully functional and no obvious abnormality is associated with microRNA processing machinery in HepG2 cells.

One interesting question raised from our observation is why TPA also down regulated SUZ12 even though only 3' UTR of EZH2 and EED's transcript carry miR-101 target sequence. Similar phenomenon has also been observed when miR-101 was ectopically overexpressed in human prostate cancer cells [17]. The authors suspected that miR-101 reduced the level of EZH2 and lead to destabilization of SUZ12. However, we cannot rule out the possibility that activation of PKCα may also down regulate SUZ12 expression in a miR-101-independent manner. We are currently investigating this possibility.

Our study provides us an excellent model to examine how expression of miR-101 is normally regulated and leads a new direction of investigation to elucidate possible defective regulatory pathway of miR-101 expression in human hepatoma cells.

## Competing interests

The authors declare that they have no competing interests.

## Authors' contributions

CWC performed experiments on Lenti-virus package, established PKC knockdown stable cell line, ERK-signaling pathway study and drafted manuscript. YH performed genome wide miRNA analysis, data analysis and drafted manuscript. KWL performed cell proliferation assay, cell cycle analysis and Western blotting analysis. LCC designed and performed luciferase assay to validate target of miR-101. HCC designed experiments, performed data analysis and drafted manuscript. SJC designed experiments, performed data analysis and drafted manuscript. CKC designed experiments, coordinated the study, and drafted manuscript. All authors read and approved the final manuscript.
